# Vestiges of underplating and assembly in the central North China Craton based on S-wave velocities

**DOI:** 10.1038/s41598-021-00756-y

**Published:** 2021-10-27

**Authors:** Haoyu Tian, Chuansong He

**Affiliations:** Institute of Geophysics, China Earthquake Administration, Beijing, 100081 China

**Keywords:** Solid Earth sciences, Physics

## Abstract

The destruction of the North China Craton (NCC) is a controversial topic among researchers. In particular, the crustal structure associated with the craton’s destruction remains unclear, even though a large number of seismic studies have been carried out in this area. To investigate the crustal structure and its dynamic implications, we perform noise tomography in the central part of the NCC. In this study, continuous vertical-component waveforms spanning one year from 112 broadband seismic stations are used to obtain the group velocity dispersion curves of Rayleigh waves at different periods, and surface wave tomography is employed to extract the Rayleigh wave group velocity distributions at 9–40 s. Finally, the S-wave velocity structure at depths of 0–60 km is determined by the inversion of pure-path dispersion data. The results show obvious differences in the crustal structure among the Western Block (WB), the Trans-North China Orogen (TNCO) and the Eastern Block (EB). The lower crust of the northern part of the EB exhibits a high-velocity S-wave anomaly, which may be related to magmatic underplating in the lower crust induced by an upwelling mantle plume. The S-wave velocity of the WB is lower than that of the TNCO in the upper and middle crust and is lower than that of both the TNCO and the EB in the lower crust. The crust of the TNCO shows higher S-wave velocities than the WB and EB in the upper and middle crust, and its overall S-wave velocity structure is clearly different from those of the WB and EB, implying that the crustal structure of the TNCO may contain vestiges of the Paleoproterozoic collision between the WB and EB and their subsequent assembly. This study marks the first time these findings are identified for the NCC.

## Introduction

**Figure 1:**
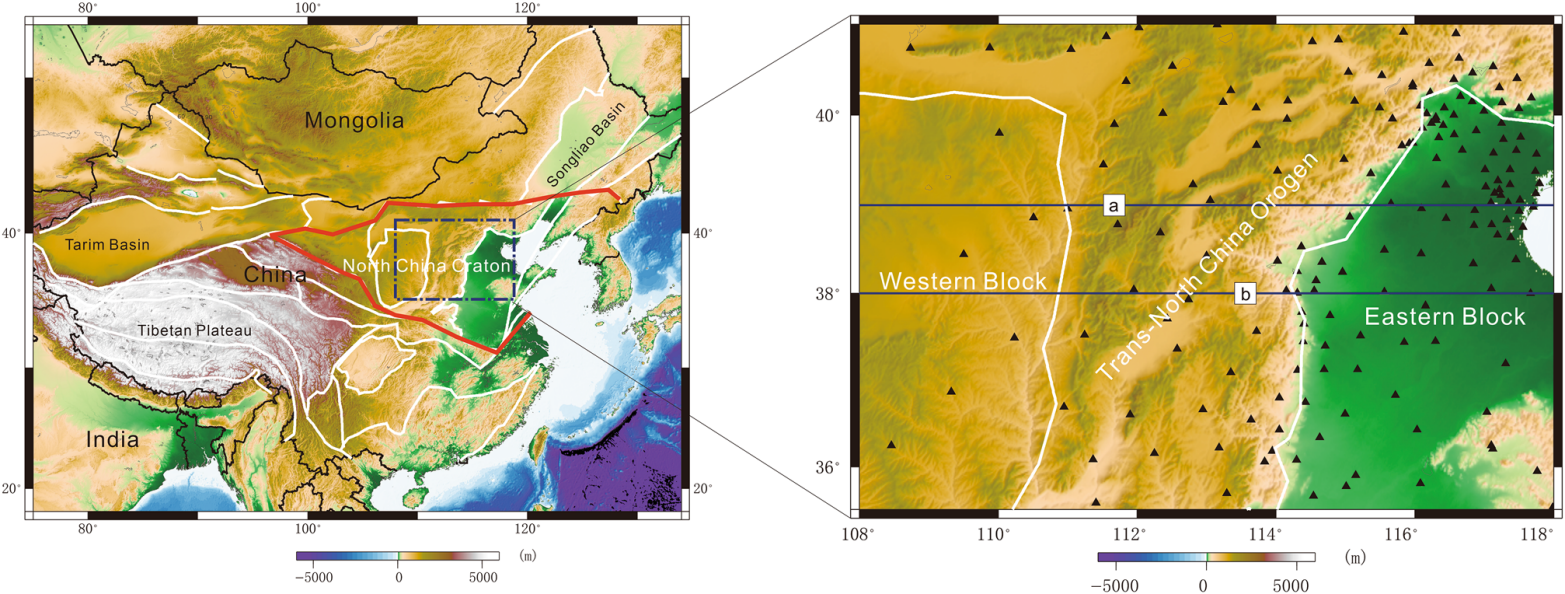
Left panel: location of the study region; red line: boundary of the NCC. Right panel: distribution of seismic stations; the black triangles indicate the seismic stations, whereas the blue lines indicate the S-wave velocity profiles. The study area is located in the central part of the NCC, including the suture zone between the EB and WB. The white lines are the boundaries of geological units (the figure was generated by Chuansong He using Generic Mapping Tools (https://www.generic-mapping-tools.org/)).

The North China Craton (NCC), one of the world's oldest cratons^1^, consists of two Precambrian terranes, the Eastern Craton (or Block) and the Western Craton (or Block) (Fig. 1). During the Paleoproterozoic, these blocks coalesced along the Trans-North China Orogen (TNCO)^2,3^. Then, the NCC remained stable for several hundred million years from the Paleoproterozoic to the late Cambrian^4^. Since the Mesozoic, however, the region has experienced multiple periods of collision among terranes and the subduction of the Pacific plate; as a consequence, the craton structure has been largely destroyed^5^. Different models have been proposed to explain the mechanism by which the craton was destroyed, such as thermal erosion generated by mantle upwelling^6–9^ and lower crustal/lithospheric delamination induced by the north–south amalgamation of terranes^10–12^.

To reveal evidence of the deep structures associated with this craton’s destruction, many seismic investigations have been carried out in this region, such as receiver function^13–19^, surface wave tomography^20,21^, P-wave tomography^11,22,23^, amplitude tomography^24^, 3-D P-wave anisotropic tomography^25^ and noise tomography^26–34^. The receiver function method has been used to define the depths of the Moho, lithosphere-asthenosphere boundary and 410 and 660 km discontinuities beneath the NCC. For example, He et al.^19^ reported that the region with a high Vp/Vs ratio of the crust corresponds well to projections on the surface of the deepening region of both the 410 km and 660 km discontinuities in the EB, which implies that mantle plume upwelling led to structural variations in the mantle transition zone and crust during the Mesozoic. P-wave tomography has been employed mainly to image the velocity structure of the upper mantle. Examples include Lei^22^ and He^11^, who similarly revealed evidence of Mesozoic mantle plume upwelling beneath the EB. Noise tomography and 3-D P-wave anisotropic tomography have been used to determine the velocity structure of the crust and uppermost mantle. For instance, Fu et al.^33^ and Tang et al.^34^ defined a low-velocity anomaly at depths of 0–20 km in the EB. However, due to the limitations of study methods or objectives, the exact crustal structure in the NCC potentially associated with deep dynamic processes, craton destruction and block assembly has not been revealed.

To finely resolve the crustal structure of this craton, we carry out noise tomography in the central part of the NCC. The results display considerable differences in the S-wave velocity structure among the WB, TNCO and EB. In particular, the distinctive S-wave velocity structure of the TNCO may be a vestige of the Paleoproterozoic assembly of cratonic blocks. We also reveal a high-velocity anomaly in the lower crust beneath the northern part of the EB that might be associated with magmatic underplating.

## Results

### Rayleigh wave velocity

**Figure 2:**
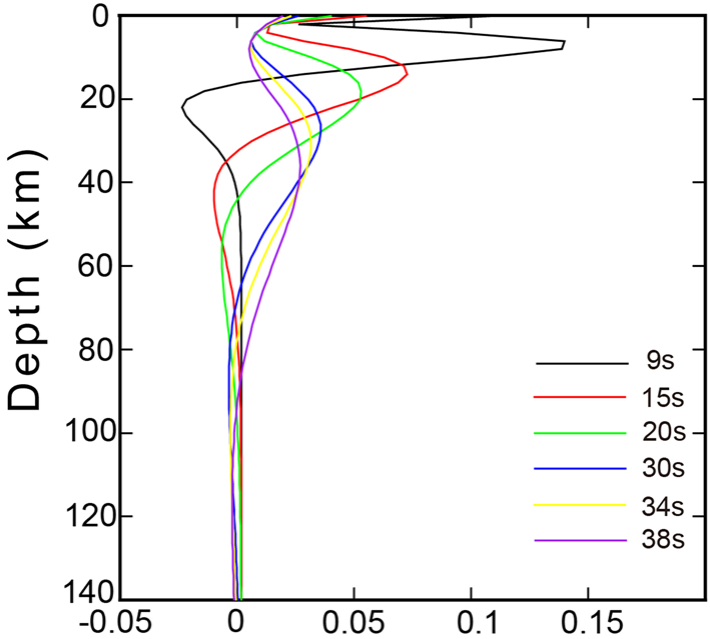
Sensitivity kernels of fundamental Rayleigh group velocities at 9 s, 15 s 20 s, 30 s, 34 s and 38 s. AK135 model^36^ is adopted for calculation, and the crustal thickness is set at 36 km [the figure was generated by Chuansong He using Generic Mapping Tools (https://www.generic-mapping-tools.org/)].

Because the group velocities at different periods have different sensitivities to the S-wave velocity in different depth ranges^35^, it is necessary to take the partial derivatives of the group velocities at different periods and obtain the sensitivity kernels of the fundamental Rayleigh wave. Our results confirm that the group velocities at several periods are well resolved at depths ranging from 6 to 42 km (Fig. 2).

**Figure 3:**
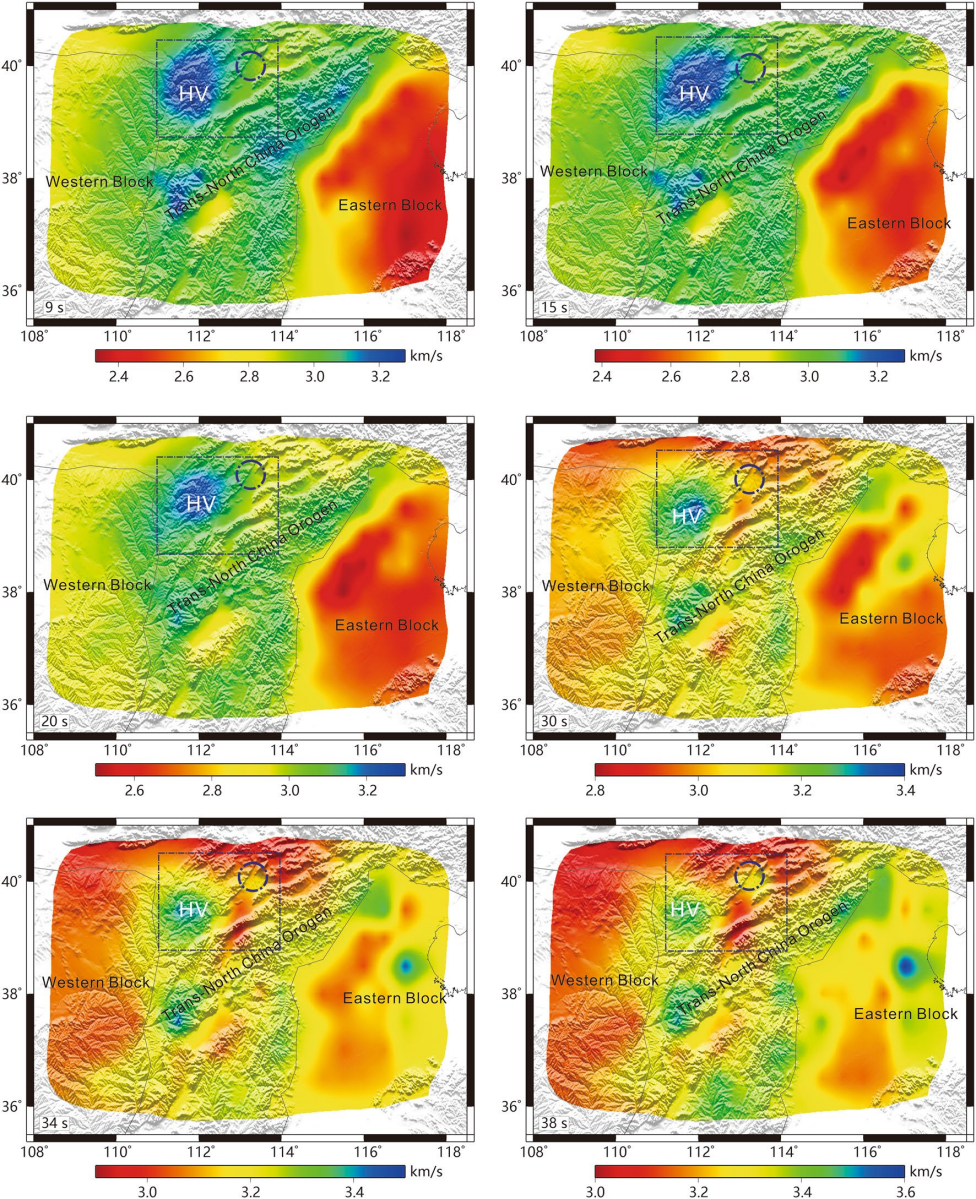
Rayleigh wave group velocities at six different periods. Blue circle: Datong volcano group. The black lines are the boundaries of geological units. Rectangle region: Cenozoic basalt region [the figure was generated by Chuansong He using Generic Mapping Tools (https://www.generic-mapping-tools.org/)].

The group velocity at a certain period is most sensitive to the S-wave velocity at 1/3 of the wavelength^37,38^, and thus, the structural differences at different depths lead to variations in the group velocity. Here, we discuss the representative group velocities at six periods (Fig. 3). The Rayleigh wave group velocity distribution at T = 9 s mainly reflects the velocity structure of the upper crust, whereas those at T = 15 s and 20 s predominantly reflect the velocity structure of the middle crust. Likewise, the Rayleigh wave group velocities at medium and long periods (30–38 s) mainly reflect the velocity structure from the lower crust to the top of the mantle (Fig. 3).

Our findings reveal that the group velocities at 9 s-30 s in the TNCO are higher than those of the terranes (EB and WB) on either side. The group velocities at 34 s-38 s in the WB are obviously lower than those in the EB (Fig. 3). In addition, the EB displays a low-velocity anomaly at a period of 9 s, which may be affected by the sedimentary layer near the surface (Fig. 3); the H–k stacking of receiver functions indicates that the sedimentary thickness in this area is between 2 and 6 km^39^. The EB is also characterized by relatively low velocities from 15 to 30 s, which might not be completely affected by the sedimentary layer (Fig. 3); indeed, this low-velocity structure may be related to a low-velocity anomaly in the middle crust. In contrast, at 34–38 s (representing the region from the lower crust to the top of the mantle), the northern part of the EB exhibits a relatively high-velocity anomaly, whereas it’s southern part shows a relatively low-velocity structure (Fig. 3).

We also detect a cylindrical high-velocity anomaly at 9–38 s (Fig. 3, HV) near Datong volcanic group^40^ and located within Cenozoic basalt region^41^ in the TNCO. Generally, basalts are generated by the upwelling of mafic magmatic material along cylindrical columns from the bottom of the lower crust^42^. This consolidated mafic magma might be responsible for the observed high-velocity structure^43^. Accordingly, we consider the high-velocity anomaly (Fig. 3, HV) to be related to magmatic upwelling from the bottom of the lower crust and the formation of the Datong volcanic group in the Cenozoic.

### S-wave velocity structure

**Figure 4:**
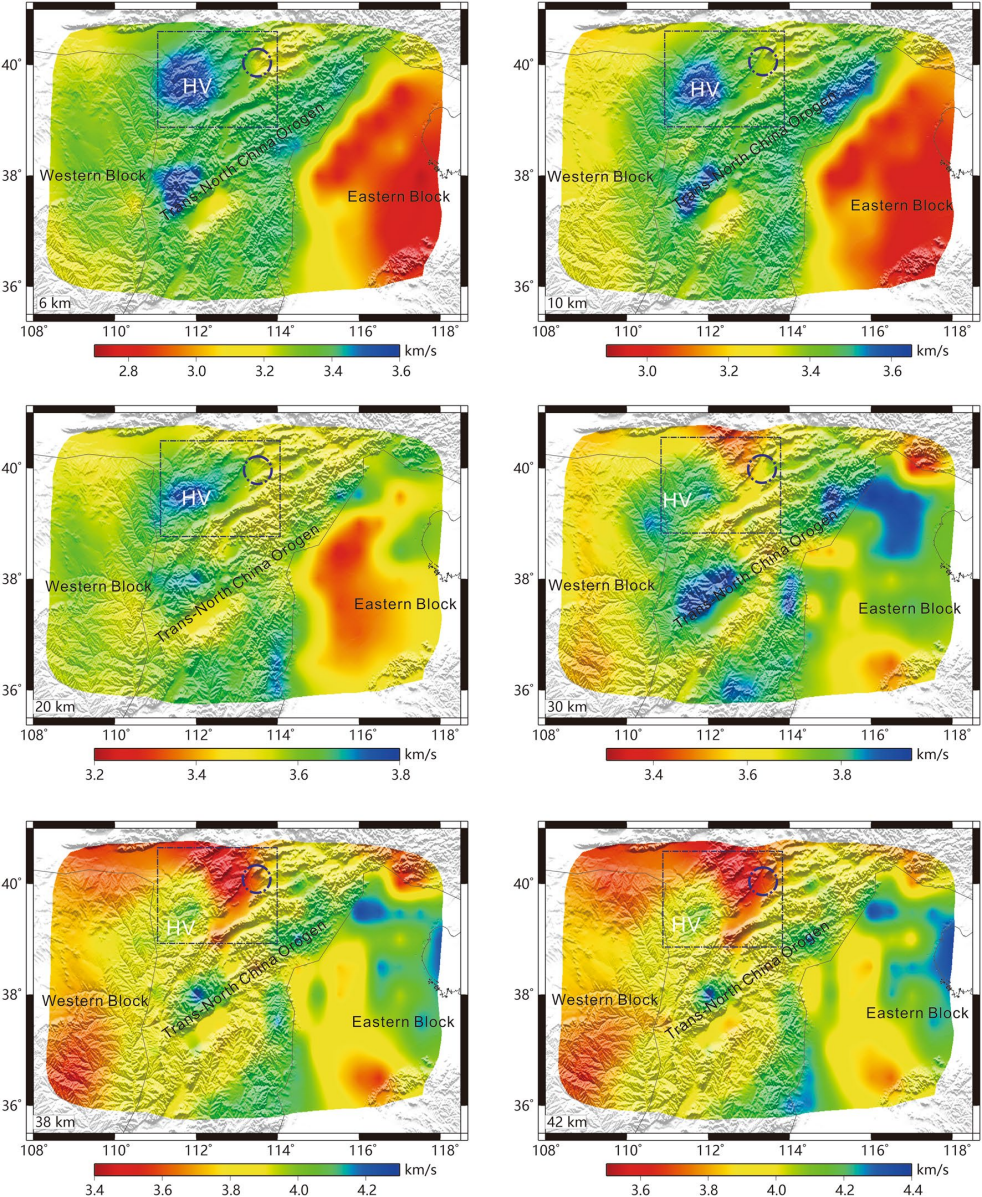
S-wave velocity distributions at depths of 6 km, 10 km, 20 km, 30 km, 38 km and 42 km. Blue circle: Datong volcanic group. The black lines are the boundaries of geological units. Rectangle region: Cenozoic basalt region [the figure was generated by Chuansong He using Generic Mapping Tools (https://www.generic-mapping-tools.org/)].

Based on the 1-D S-wave velocity structure at each grid node obtained in this study, we construct a 3-D S-wave velocity model in the depth range from 6 to 42 km (Fig. 4). The results show that the S-wave velocity of the TNCO is higher than those of the EB and WB at depths from 6 to 20 km (Fig. 4). Furthermore, the S-wave velocity in the WB is lower than that in the EB at depths of 38 and 42 km (Fig. 4). In contrast, beneath the EB, a low-velocity anomaly is observed at a depth of 6 km, and a relatively low-velocity anomaly is noted at depths of 10–20 km (Fig. 4). At depths of 38–42 km, a high-velocity anomaly is detected in the northern part of the EB, whereas a low-velocity structure is revealed in the southern part of the EB (Fig. 4). In addition, the Surface wave velocity model also reveals a cylindrical high-velocity anomaly at depths from 6 to 42 km near the Datong volcanic group within the TNCO (Fig. 4, HV). The above results are basically consistent with the group velocity distribution (Fig. 3).

**Figure 5:**
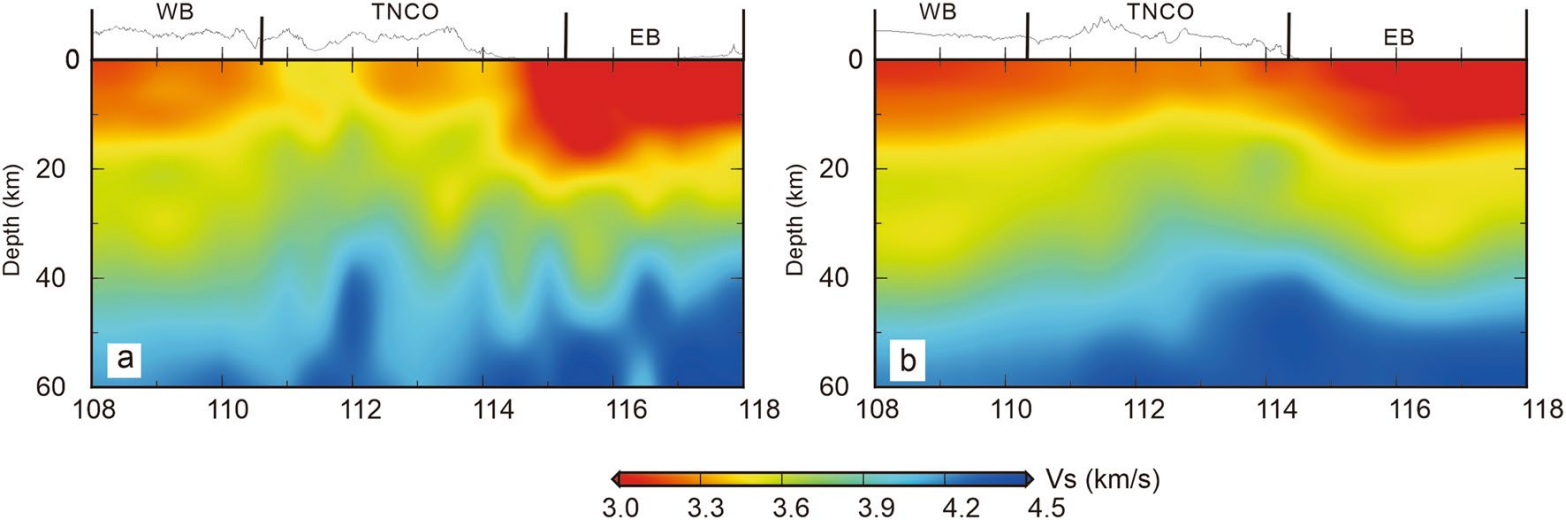
S-wave velocity profiles [the figure was generated by Chuansong He using Generic Mapping Tools (https://www.generic-mapping-tools.org/)].

We also establish two S-wave velocity profiles across the study region (Fig. 5; the profile locations are indicated in Fig. 1). The profiles illustrate that the S-wave velocity is between 3.8 and 4.2 km/s at depths of 40–45 km in the WB, whereas the S-wave velocity is between 3.8 and 4.2 km/s at depths of 35–42 km in the EB, which might be related to the Moho depth. These results are partly consistent with the results of the H–k stacking of receiver functions (Fig. S1)^19^. Due to the limitation of surface waves in resolving the Moho, the crustal thickness inferred from the surface wave tomography is greater than that inferred from the H–k stacking of the receiver functions. However, the S-wave structure basically exhibits a trend of gradual crustal thickening from east to west.

## Discussion

He et al.^19^ determined the distributions of the crustal thickness and Vp/Vs ratio in the NCC by performing H–k stacking of receiver functions. Their results reflected a high Vp/Vp ratio (bulk crustal Vp/Vs ratio > 1.76, Fig. S1, red ellipse in the right panel) in the northern NCC, indicating that the Vp/Vs ratio in the lower crust might be greater than 1.78 or 1.80^19^. In general, magmatic underplating can lead to high Vp/Vs ratios^43,44^ and high-velocity anomalies in the lower crust^43^. This study reveals that the EB contains a high-velocity anomaly at depths of 38 km and 42 km, either at the bottom of the lower crust or at the top of the mantle (Fig. S1, left panel; the crustal thickness in the EB is between 32 and 38 km). This supports the occurrence of deep magmatic underplating in this area. Due to the uneven distribution of seismic stations in the EB (Fig. S1, black triangles), this region with a high Vp/Vs ratio in the EB is not completely resolved. Nevertheless, the areas with a considerably high Vp/Vs ratio correspond to the high-velocity anomaly in the lower crust (Fig. S1, Fig. 4).

In general, an upwelling mantle plume plays a key role in the occurrence of underplating in the lower crust^45^. He^11^ reported a mushroom-like low-velocity structure (potentially a Mesozoic vestige of an upwelling mantle plume beneath the EB) by using P-wave teleseismic tomography (Fig. S2); this region corresponds to the area in the EB where the lower crust may be underplated. This finding implies that an upwelling mantle plume affected the crustal and lithospheric structure of the NCC or contributed to the destruction of the NCC.

He et al.^19^ also revealed a region with a high Vp/Vs ratio in the WB (Fig. S1, blue ellipse in the right panel). Our results identified in this study indicate relatively low S-wave velocities at depths of 38 km and 42 km in the lower crust of the WB (Fig. 4), which rules out the occurrence of magmatic underplating in the lower crust therein. Recent crustal structure studies indicate that the average crustal S-wave velocity of the Precambrian crust is ~ 3.6 km/s, which is higher than that of the crust after the Paleozoic^46,47^. Seismic studies indicate that the Vp/Vs ratio increases with the age of the crust; for example, Precambrian shields and ancient cratons tend to exhibit high Vp/Vs ratios^43^; moreover, the average crustal S-wave velocity in the WB identified in this study is only ~ 3.6 km/s. Therefore, the high Vp/Vs ratios in the WB might indicate that the block retains remnants of ancient crust, implying some preservation of the cratonic structure in the WB.

TNCO is an N-S-oriented linear belt that formed as a consequence of the collision between EB and WB, culminating in the final assembly of the NCC ca. 2.1–1.8 Ga^48^. Along the linear TNCO, high-pressure assemblages can be traced for more than 700 km^49^, reflecting continental collision and extrusion environments^50^. The compressional structure can lead to bulk high-velocity anomaly in the crust^51^. Both the group velocities and the S-wave velocities in the crust indicate that the TNCO is a relatively high-velocity terrane, which might reflect its extrusion/compressional environments induced by the collision/assembly between the EB and WB. The velocity structure also indicate that the TNCO is an independent geological unit that connects the EB and WB (Figs. 3, 4). These imply that the TNCO may contain vestiges of the Paleoproterozoic collision between the EB and WB and their subsequent assembly.

Recently, the high S-wave velocity have also been defined by surface wave tomography in stable collisional orogens^52,53^ such as the Taiwan collisional orogen and the southern Zagros collisional orogen of Iran. On the contrary, crustal low-velocity zones have been reported in active orogens such as the Andes and the Himalayas^54^.

## Conclusions

This study reveals that the group velocities and S-wave velocities of the EB and WB of the NCC and those of the TNCO are quite different. In the WB, the relatively low S-wave velocity and high Vp/Vs ratio in the lower crust may represent ancient lower crust, which implies that its cratonic structure has not been destroyed. In contrast, in the northern part of the EB, the high S-wave velocity and high Vp/Vs ratio in the lower crust may reflect magmatic underplating along the base of the Moho induced by an upwelling mantle plume, which implies that an ascending mantle plume may have contributed to the destruction of the NCC. Furthermore, we suggest that the S-wave velocity structure of the TNCO contains vestiges of the collision between the WB and EB in the Paleoproterozoic and their subsequent assembly.

## Data and method

### Data and data processing

Based on a direct cross-correlation of the continuous background noise between two seismic stations, we can perform high-resolution seismic imaging in the crust and upper mantle^55–61^.

In this study, the continuous vertical component waveforms recorded by 112 seismic stations from January 2018 to December 2018 are collected from the Data Management Center of the China National Seismic Network. Based on the method of Bensen et al.^59^, we resampled the continuous waveforms to 5 Hz and cut the data from 0 to 24 h. Other steps involve synchronizing the clock, removing the instrument response, bandpass filtering (4–50 s period), normalization in the time domain, and spectral whitening. Finally, the daily waveform at each seismic station is correlated with those at all other seismic stations, and the daily results at each seismic station are stacked to produce the final cross-correlation results.

The surface wave signals coming from opposite directions along the path linking two seismic stations can be inferred from the resulting cross-correlations. Due to the inhomogeneous distribution of ambient noise sources, the cross-correlations might be asymmetrical. To enhance the signal-to-noise ratio of the surface waves and simplify the data analysis, each cross-correlation is separated into negative and positive lag components, and then the two components are added to form the so-called symmetric component. The following analysis is performed exclusively on the symmetric signals.

Based on multiple filtering techniques^62,63^, we manually pick the group velocity dispersion curve with Computer Programs in Seismology (CPS) software^64^. Assuming there are n stations, we can extract empirical Green’s functions on n(n-1)/2 paths. To ensure reliable results, we implement quality control on the dispersion curve.

An empirical Green’s function is acceptable if the interstation distance is at least 3 times the wavelength at a given period, and acceptable autopicked group velocities were chosen according to the wavelength and signal-to-noise ratio^58,59^. Finally, we select a total of 1774 dispersion curves for the station pairs from the 6216 Rayleigh wave waveform data (see Fig. S3). A plot of the ray paths used for surface wave imaging at different periods shows that the number of rays at different periods is relatively uniform (Fig. S4).

### Rayleigh wave velocity and S-wave velocity inversion

In the process of surface wave tomography, a generalized 2-D linear inversion procedure is used to construct the distribution of the group velocity^65,66^, which is a generalization to 2-D inferred from the classic 1-D method of Backus and Gilbert^67^. In the inversion process, a 0.5° × 0.5° lateral grid is designed, and a damping parameter of α = 0.2 is selected according to different damping parameter (α = 0.1, 0.2 and 0.3) tests, which controls the tradeoff between the fit to the data and the smoothness of the resulting group velocity maps and yields relatively smooth maps with small fitting errors.

From the Rayleigh wave group velocity obtained by the above inversion approach, the dispersion curves of the group velocity at each grid node are extracted. The 1-D S-wave velocity structure at each grid node is then inverted^68^, as shown in Fig. S5, and the velocities between the nodes are interpolated linearly. In this way, we construct a 3-D model of the S-wave velocity structure.

A constant S-wave velocity of 4.38 km/s from the surface to a depth of 90 km is designed for the initial model, which is divided into 2 km layers. This initial model avoids the artificial addition of low-velocity zones. In the inversion process, a fixed Vp/Vs ratio of 1.732 is adopted, and the density is obtained from the P-wave velocity^69^.

### Resolution analysis

Generally, a checkerboard resolution test (CRT) is conducted to analyse the resolution and estimate the error of the results. In this study, another reliable technique is used to evaluate the resolution; that is, we estimate the spatially averaged kernel at each grid node in different directions or average area^70^, which can be approximated by an ellipse centred at a point in the 2-D tomography. Based the smallest and largest axes of the ellipse, we can calculate smallest and largest value of the ellipse as well as averaging area of the ellipse.

The resolution radius distribution shows that the minimum resolution radius can reach 50 km, whereas for most of the study area, the resolution radius can reach 200 km (Fig. S6). In this region, the spatially averaged resolution radius is between 0 and 200 km, and the resolution radius is completely within the smoothing radius allowed by the model parameters. According to the resolution detection results, we consider that the inversion results in our study region are reliable.

## Supplementary Information

Supplementary Figures.

## Data Availability

The data of the cross-correlation function involved in the noise tomography can be accessed via https://doi.org/10.5281/zenodo.4971734.
