# Supplementary material for: Vestiges of underplating and assembly in the central North China Craton based on S-wave velocities
Source: Sci Rep. 2021 Oct 27;11:21218. doi: 10.1038/s41598-021-00756-y (PMC8551183; doi:10.1038/s41598-021-00756-y)
Supplement: Supplementary file 1 — Supplementary Figures. [file 41598_2021_756_MOESM1_ESM.docx]

**Vestiges of Underplating and Assembly in the Central North China Craton Based on S-wave Velocities**

Haoyu Tian, Chuansong He

Institute of Geophysics, China Earthquake Administration, Beijing 100081, China

Corresponding author: Chuansong He, email: hechuansong@aliyun.com


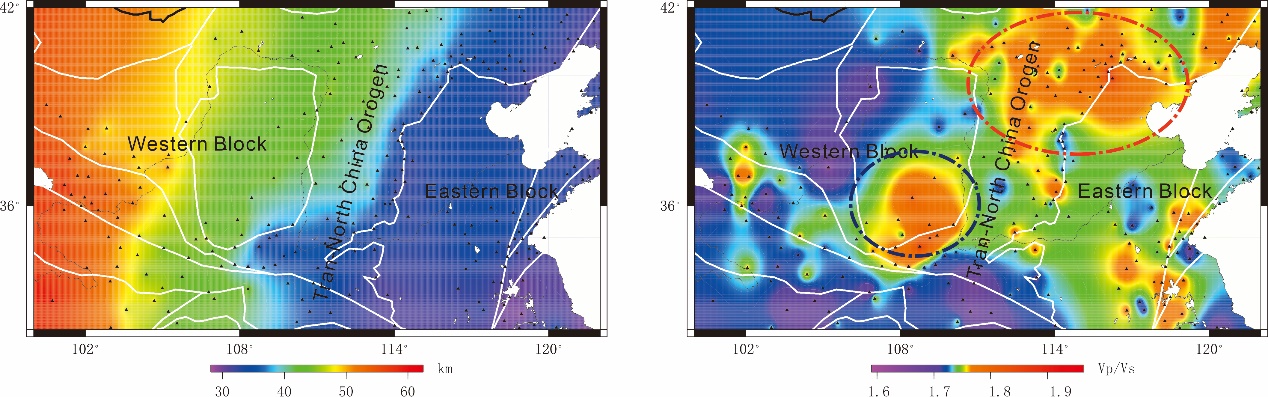


**Figure S1**. Left panel: crustal thickness distribution; right panel: Vp/Vs ratio distribution in the NCC (He et al., 2015). Black triangle: seismic station (the figure was generated by Chuansong He using the Generic Mapping Tools (https://www.generic-mapping-tools.org/)).

**
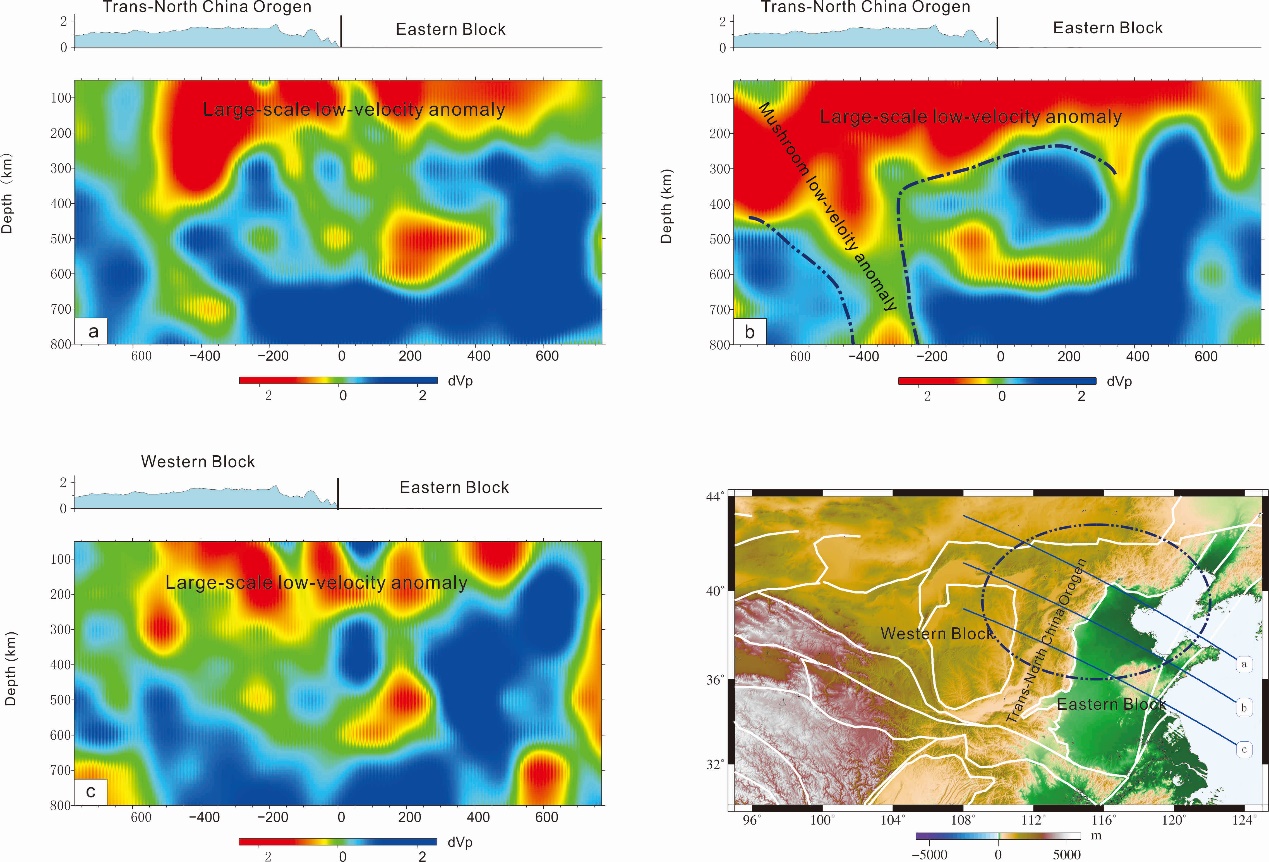
**

**Figure S2**. P-wave velocity perturbation profile (He, 2020), b: Low-velocity anomaly with a mushroom shape (the figure was generated by Chuansong He using the Generic Mapping Tools (https://www.generic-mapping-tools.org/)).


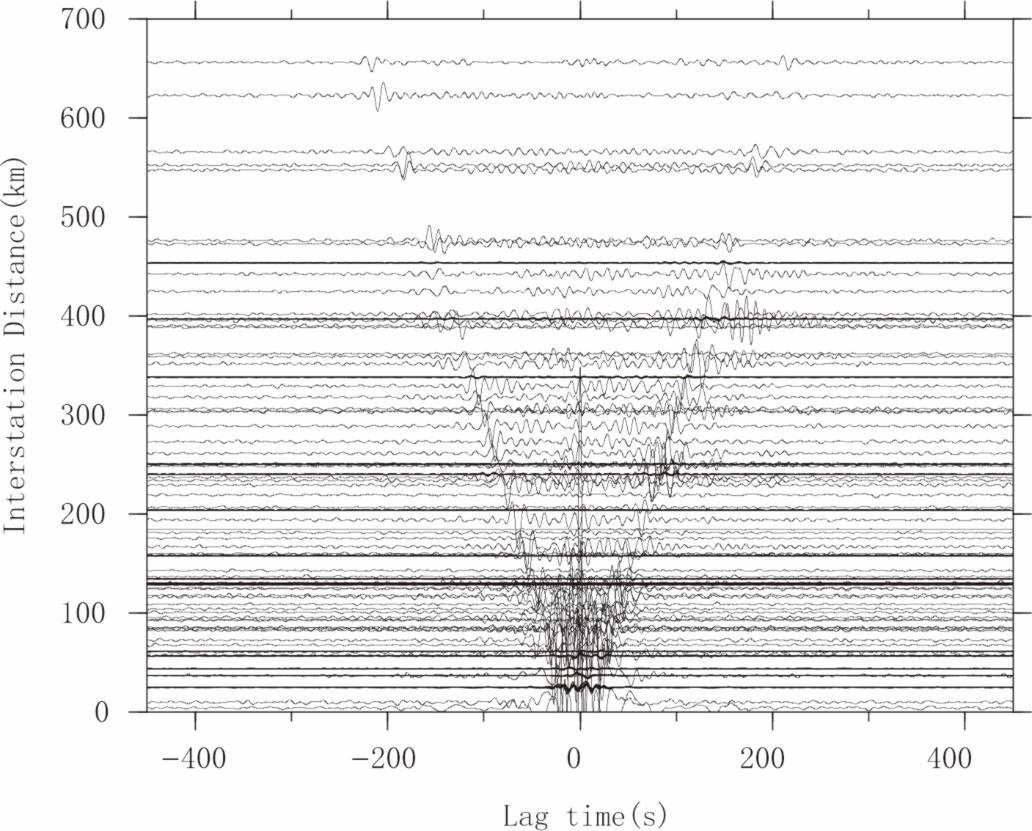


**Figure S3**. Example of the correlation of one-year data from the ZKD seismic station related to other seismic stations with periods from 5 to 50 s (the figure was generated by Chuansong He using the Generic Mapping Tools (https://www.generic-mapping-tools.org/)).


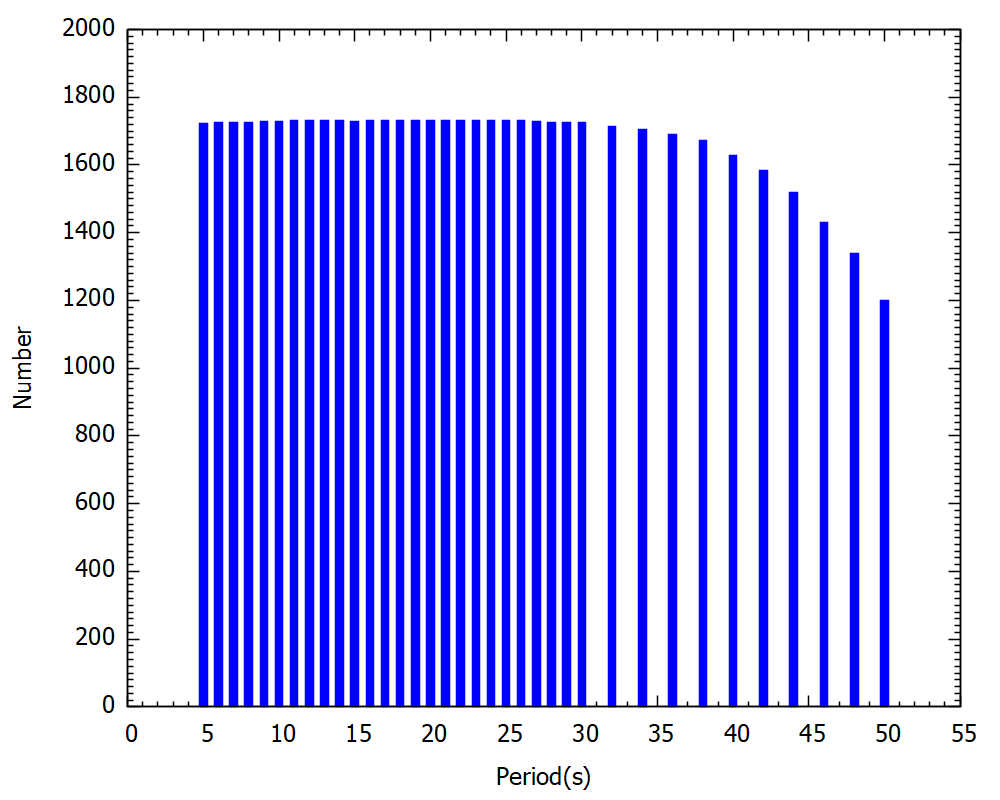


**Figure S4**. The number of group velocity dispersion curves at different periods (the figure was generated by Chuansong He using the Generic Mapping Tools (<https://www.generic-mapping-tools.org/)>).


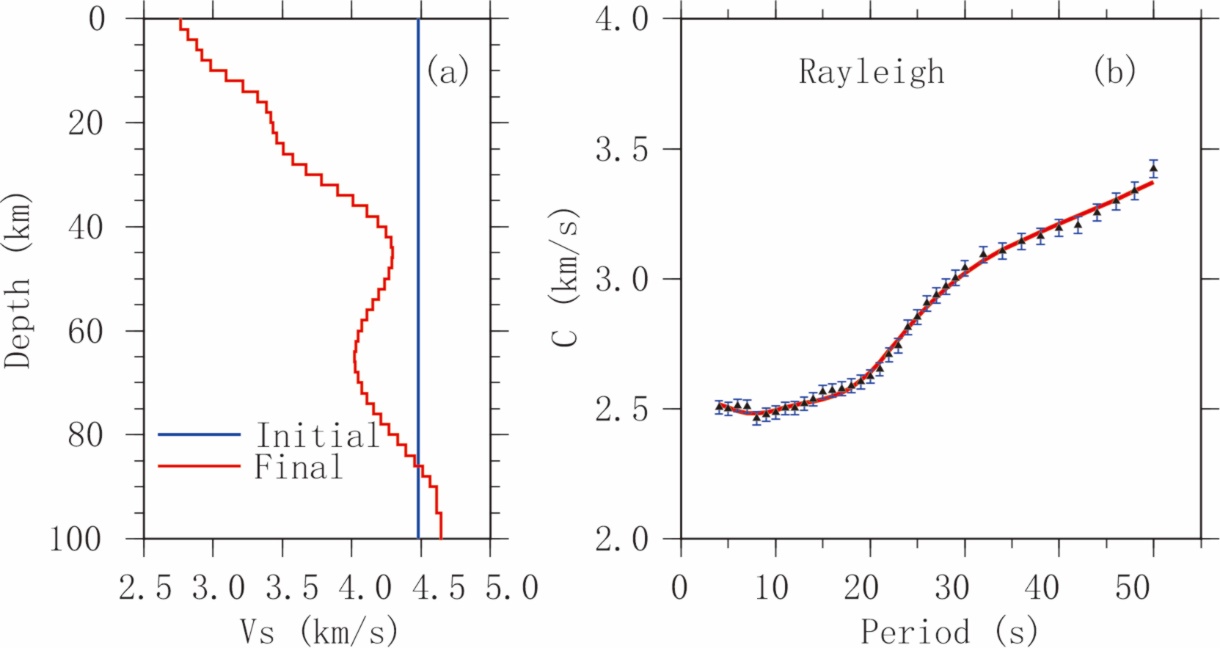


**Figure S5**. An example to illustrate the process of inverting the S-wave velocity from the dispersion curve at a node (116.5°E, 38°N). Uncertainties in phase velocity measures are evaluated from empirical Green Functions with different stacking durations. Uncertainties in Vs inversions are evaluated through one standard deviation of the Vs models inverted from randomly generated 100 initial models. The tests, which report the uncertainty of the inverted crustal Vs model is 0.05-0.15 km/s depending on depth, demonstrate that our inversion results are quite stable. The red solid line of the right panel represents the theoretical group velocity dispersion generated by the final S-wave velocity model and obtained from the inversion. The black triangles in the right panel indicate the dispersion of group velocity observations. The blue dashed line in the left panel represents the initial velocity model, whereas the red solid line represents the final S-wave velocity model obtained by the inversion (the figure was generated by Chuansong He using the Generic Mapping Tools (<https://www.generic-mapping-tools.org/)>).


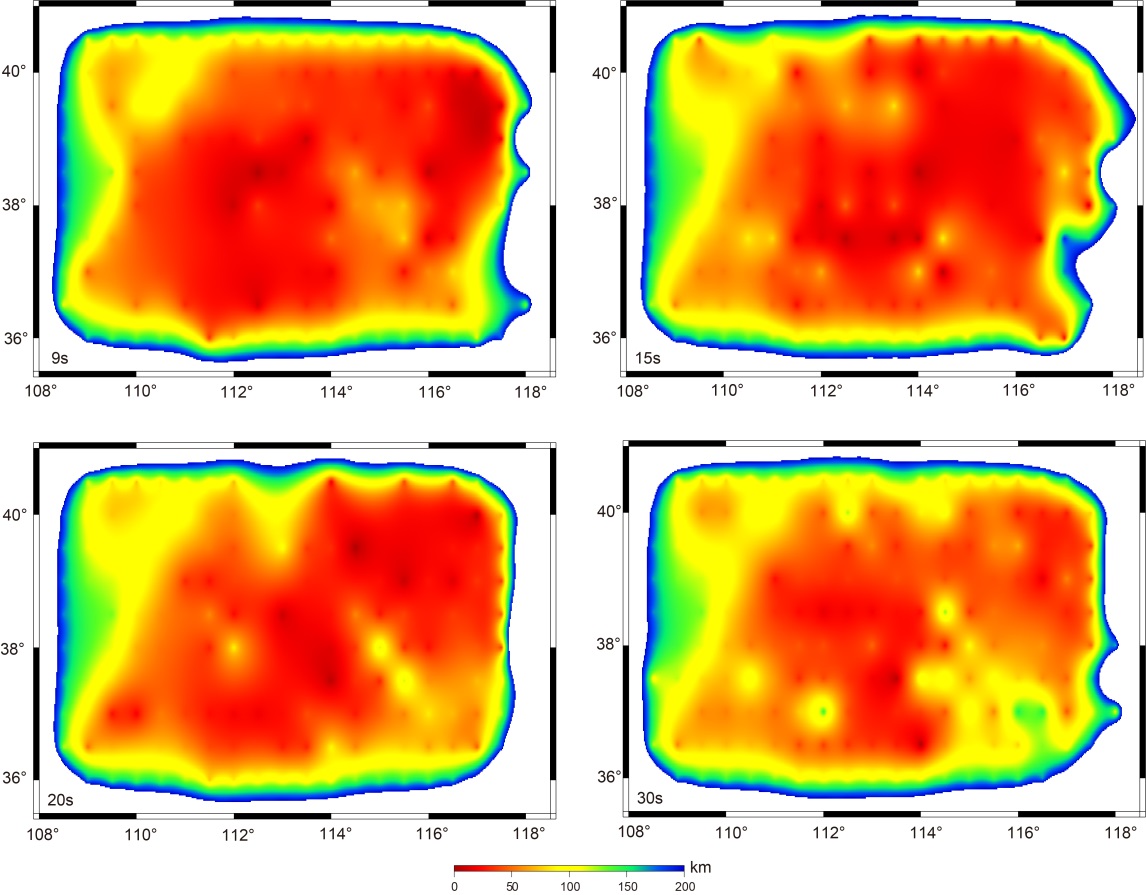


**Figure S6**. The distribution of the spatial average resolution radius at different periods. The color scale at the bottom shows the value of the resolution radius (the figure was generated by Chuansong He using the Generic Mapping Tools (<https://www.generic-mapping-tools.org/)>).
